# From prompt to platform: an agentic AI workflow for healthcare simulation scenario design

**DOI:** 10.1186/s41077-025-00357-z

**Published:** 2025-05-16

**Authors:** Federico Lorenzo Barra, Giovanna Rodella, Alessandro Costa, Antonio Scalogna, Luca Carenzo, Alice Monzani, Francesco Della Corte

**Affiliations:** 1 Department of Anesthesiology and Intensive Care Medicine, Azienda Ospedaliero-Universitaria “Maggiore Della Carità”, Novara, Italy; 2 SIMNOVA-Interdepartmental Center for Innovative Learning and Simulation in Medicine and Allied Health Professions, University of Piemonte Orientale, Novara, Italy; 3 Department of Anesthesiology and Intensive Care Medicine, Presidio Ospedaliero SS. Trinità, ASL 13, Borgomanero, Italy; 4 Department of Anesthesia and Intensive Care Medicine, IRCCS Humanitas Research Hospital, Rozzano, Italy; 5 Division of Pediatrics, Department of Health Sciences, University of Piemonte Orientale, Novara, Italy

**Keywords:** Artificial intelligence, AI, Agentic workflow, Scenario design, Healthcare simulation, Automation, N8n, ChatGPT, INACSL standards, ASPiH standards

## Abstract

Healthcare simulation scenario design remains a resource-intensive process, demanding significant time and expertise from educators. This article presents an innovative AI-driven agentic workflow for healthcare simulation scenario development, bridging technical capability with pedagogical effectiveness. The system evolved from an initial ChatGPT-based prototype to a sophisticated platform implementation utilizing multiple specialized AI agents. Each agent addresses specific sub-tasks, including objective formulation, patient narrative generation, diagnostic data creation, and debriefing point development. The workflow employs advanced AI methodologies including decomposition, prompt chaining, parallelization, retrieval-augmented generation, and iterative refinement, all orchestrated through a user-friendly conversational interface. Critical to implementation was the demonstration that healthcare professionals with modest technical skills could develop these complex workflows without specialized AI expertise. The system ensures consistent adherence to established simulation guidelines, including INACSL Standards of Best Practice and ASPiH Standards Framework, while significantly reducing scenario development time by approximately 70–80%. Designed for broad applicability across diverse clinical settings and learner levels, the workflow incorporates multilingual capabilities for global application. Potential pitfalls include the necessity for rigorous review of AI-generated content and awareness of bias in model outputs. Key lessons learned emphasize interdisciplinary collaboration, systematic prompt refinement, essential human oversight, and the democratization of AI tools in healthcare education. This innovation demonstrates how sophisticated agentic AI implementations can transform healthcare simulation through enhanced efficiency, consistency, and accessibility without sacrificing pedagogical integrity.

## Introduction

The cornerstone of effective healthcare simulation lies in meticulously crafted scenarios that provide realistic, controlled environments for learners to develop clinical skills and practice decision-making [1–3]. Traditionally, the creation of these high-fidelity scenarios has been a resource-intensive process, demanding significant time and expertise from simulation educators. The often-quoted figure of 24 h of preparation for a 10–20 min scenario underscores the substantial investment required [1, 4]. The recent advent of advanced large language models (LLMs), such as ChatGPT, has sparked considerable interest in their potential to revolutionize this process [5–8]. Early explorations suggest that LLMs can contribute to streamlining scenario development, potentially offering insights that conventional methods might miss. However, concerns regarding the accuracy, relevance, and structural coherence of AI-generated content have also been raised, necessitating careful consideration and human oversight [9].

This article chronicles the development and implementation of an innovative, AI-powered, agentic workflow for healthcare simulation scenario design in SIMNOVA Simulation Center in Novara, Italy. The project evolved from an initial prototype based on a structured ChatGPT interface to a fully automated system leveraging “n8n,” a no-code open-source platform. The narrative details the development process, the underlying rationale, the scope of potential applications, and the critical lessons learned. The overarching objective is to provide simulation educators with a comprehensive understanding of how AI-driven approaches can enhance efficiency and consistency in scenario design while ensuring rigorous adherence to established best-practice standards.

## Aim and objectives

The primary aim of this innovation was to develop an AI-powered system capable of significantly reducing the time and effort required to create high-quality, standards-compliant healthcare simulation scenarios. This reduction in development time was hypothesized to free up valuable educator resources, allowing for greater focus on learner interaction and debriefing [10].

Beyond simply accelerating the process, the project aimed to achieve several specific objectives. First, the system needed to automate the generation of core scenario components, including learning objectives, a detailed patient narrative, relevant diagnostic data, and comprehensive debriefing points. Second, it was essential that the system’s outputs were consistently aligned with established simulation design standards, specifically the INACSL Standards of Best Practice: Simulation Design and the ASPiH Standards Framework for Simulation-Based Education [11, 12]. Third, user-friendliness was paramount; the workflow needed to be accessible to educators with varying levels of technical expertise, not requiring specialized programming skills. Fourth, the system was designed for adaptability, supporting customization for a wide range of clinical settings and learner levels, from undergraduate nursing students to experienced medical professionals. Fifth, to enhance its global applicability, the system incorporated multilingual scenario generation capabilities. Finally, the development process prioritized addressing potential issues of factual accuracy, bias in AI outputs, and ethical considerations related to intellectual property and patient privacy.

## Target group

The primary target group for this innovation encompasses a broad spectrum of individuals involved in healthcare simulation. This includes simulation educators across various disciplines (nursing, medicine, allied health professions), simulation center directors and technical staff responsible for scenario implementation, and curriculum developers seeking to integrate simulation-based learning into their programs. Furthermore, the system is particularly relevant for institutions aiming to expand their simulation offerings and for educators working in resource-constrained settings where time and expertise may be limited. The system’s accessibility aims to lower the barrier to entry for those new to simulation scenario design, while also providing advanced capabilities for experienced simulationists.

## Development process

**Fig. 1:**
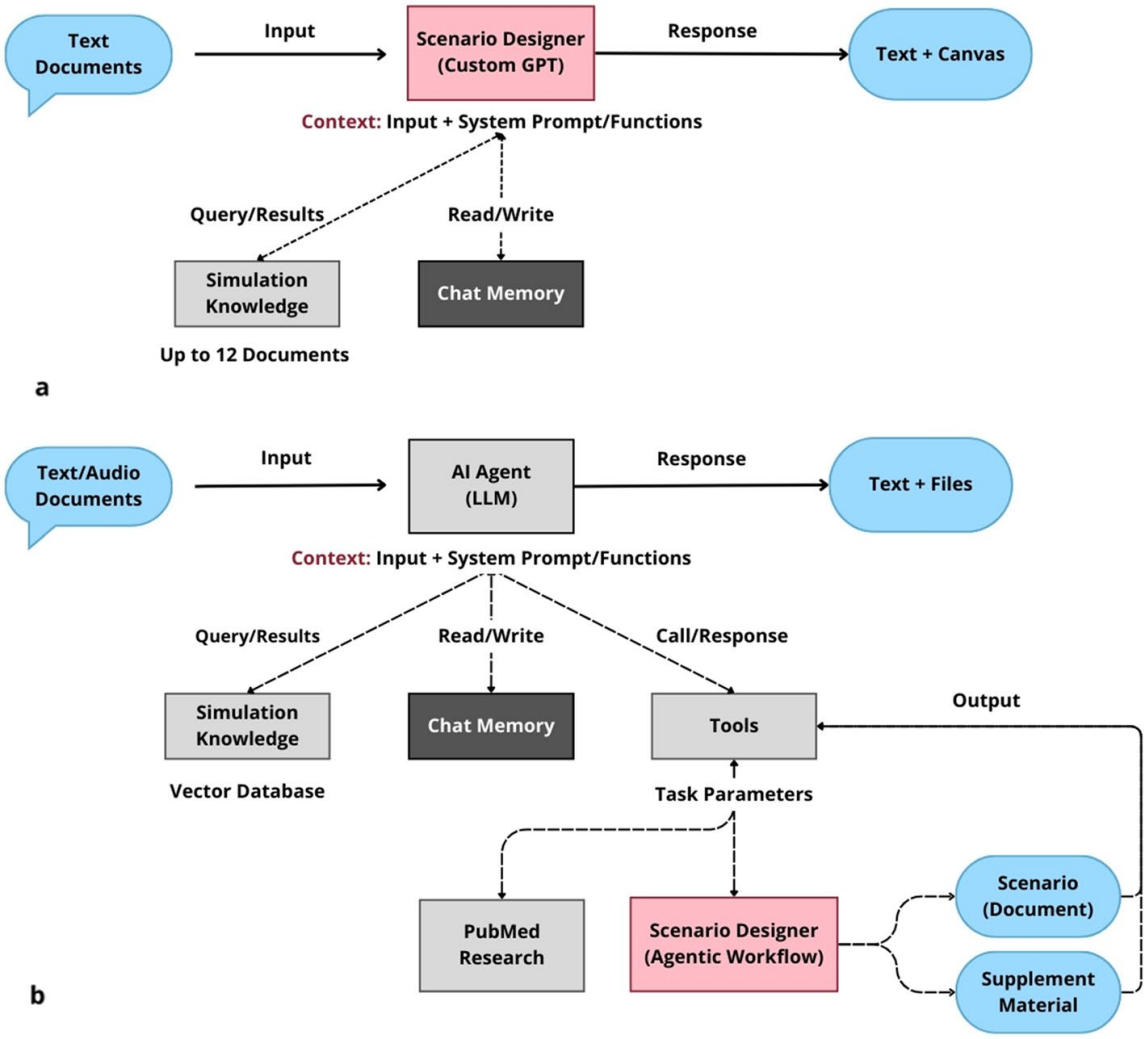
Evolution from ChatGPT-based prototype to agentic AI workflow. **a** Initial ChatGPT-based scenario designer. A structured, custom interface powered by ChatGPT, specifically trained with simulation content and scenario templates. Educators interact with the AI via a chat-based interface within the ChatGPT platform, guiding the model through a series of prompts to generate scenario elements. The LLM generates an editable Canvas that allows users’ refinements and finalization. Limitations are text-based outputs, limited knowledge retrieval, and lack the capacity for external system integration or automated visual aid generation. **b** Advanced agentic workflow in n8n. Featuring an AI agent that autonomously makes decisions (research context, create scenario) and takes actions without constant human oversight. Educators still interact with the system via a chat-like interface but with greatly expanded accessibility. This interaction can occur through various channels, including local or web-based pages and mainstream messaging systems. The knowledge is an unlimited vector database, utilizes diverse tools, and seamlessly integrates external APIs. The key action is the generation of formatted files containing the complete simulation scenario and supplementary materials, such as arterial blood gases (ABGs), lab exam results, and imaging findings, delivered directly to the user, that has the task to review the material and finalize the scenario

The development of the AI-assisted scenario design system unfolded in two distinct, yet interconnected, phases (Fig. 1).

### Phase 1: the initial ChatGPT-based scenario designer

Initially, we focused on creating what OpenAI refers to as GPTs (generalized pre-trained models). These are structured, custom, interactive interfaces powered by ChatGPT (OpenAI, USA), tailored for specific tasks or topics [13]. This implementation took the concrete form of a publicly accessible custom GPT titled “Scenario Designer,” [14] (Fig. 1a) specifically trained with comprehensive simulation scenario templates and specialized materials for healthcare simulation scenario creation. Rather than relying on free-form interaction with the LLM, this customized application guided users through a methodically structured series of prompts, systematically eliciting key parameters such as the clinical case type, target learner demographic, and specific learning objectives.

This structured approach, informed by established scenario design principles [11, 12], ensured that the AI-generated content adhered to standardized formats and comprehensively addressed all critical elements required for effective simulation scenarios. The system sequentially generated a short text for each discrete section of the scenario, allowing educators to review and refine the AI’s output at each developmental stage. This iterative process proved crucial for maintaining rigorous human oversight and ensuring clinical and pedagogical accuracy.

The Custom GPT underwent significant evolution in December 2024 when it was enhanced to leverage ChatGPT’s Canvas functionality [15]. This technical advancement enabled users to modify the scenario structure directly within the web interface, facilitating a more dynamic and responsive design process. The Canvas implementation allowed for real-time visualization and manipulation of the scenario framework, enhancing user engagement and streamlining the refinement process.

Throughout development, simulation educators served as key informants and stakeholders, providing feedback and modifying the system prompt and template’s structure and the quality of the AI-generated content. This collaborative approach ensured that the technological implementation remained firmly grounded in evidence-based simulation pedagogy. The phase was conceptually anchored in the understanding that LLMs could significantly accelerate the drafting of scenario components, effectively reducing development time and allowing educators to allocate their expertise toward refining the scenario’s nuances and critical pedagogical aspects [10].

Despite its advantages, this implementation demonstrated several inherent limitations. First, the system remained entirely contained within the ChatGPT environment, restricting its integration with external systems and workflows, and limiting its content knowledge to a maximum of 12 documents. Second, it relied exclusively on the OpenAI models, preventing exploration of alternative LLM architectures potentially better suited for specific aspects of scenario design. Third, the system lacked connectivity with document creation tools, necessitating manual export and formatting of generated content. Finally, its outputs were limited to text, precluding the automatic generation of visual elements that could enhance scenario fidelity and learner engagement.

### Phase 2: from chatbot to agent: building an AI workflow with n8n

These limitations ultimately necessitated the transition from a guided chatbot interaction to a fully agentic AI workflow. An *AI agent*, in this context, represents an autonomous system capable of perceiving its environment, making independent decisions, and taking actions to accomplish specific objectives without constant human intervention. Unlike conventional chatbots that primarily respond to direct prompts within a single conversation, agents can maintain persistent state, execute sequences of operations across multiple systems, and adaptively respond to changing conditions over time. This agentic approach enables a significant expansion of functionality, allowing the AI to perform complex tasks such as accessing databases, triggering simulations, evaluating outcomes, and coordinating multiple information streams, all while maintaining coherence within the overarching workflow framework. An automated agentic workflow with AI large language models (LLMs) constitutes a structured sequence of processes where LLMs function as integral components within a broader orchestrated system. Such workflows systematically coordinate the execution of predefined tasks, leveraging LLMs’ linguistic and reasoning capabilities at strategic intervention points while maintaining procedural consistency through formalized data pathways. These workflows enable the creation of robust systems capable of processing information, making determinations, and initiating subsequent actions according to predetermined logical frameworks without requiring continuous human oversight for routine operational decisions.

The n8n platform (n8n Software, Germany) was selected for several critical reasons that aligned with the project’s objectives and constraints. First, its open-source nature eliminated licensing costs and provided full transparency into the workflow’s operations. Second, its ability to self-host offered simulation centers complete control over their data and infrastructure, addressing potential privacy concerns when working with sensitive clinical scenarios. Third, n8n’s flexibility in integrating with diverse LLM providers (including OpenAI, Anthropic, Google and others) prevented vendor lock-in and allowed for adaptation as new models emerged.

A critical aspect of this phase was that the entire development process was executed by the lead author, a Critical Care physician and Healthcare Simulation Educator with basic coding knowledge but no specialized AI expertise. This approach demonstrated that healthcare simulation educators with modest technical skills can successfully implement sophisticated AI workflows without requiring dedicated AI specialists or software engineers. This democratization of AI implementation is particularly significant for simulation centers operating with limited technical resources but possessing motivated staff willing to explore innovative approaches.

The workflow leverages a strategic combination of AI models to optimize both performance and cost-effectiveness. The system primarily utilizes GPT-4o for tool-calling agents that require specialized capabilities, Gemini 2.0 Flash and Pro for the main workflow components due to their extensive context token capacity and cost-free availability (significantly reducing operational expenses), and Anthropic Claude 3.7 Sonnet for reviewing and final editing to ensure high-quality outputs. This thoughtful model selection exemplifies how simulation centers can implement sophisticated AI workflows while managing costs effectively. The authors also tested a complete local LLMs system powered by Qwen QWQ 32B (Alibaba, China) and Mistral Small 3 (Mistral AI, France), achieving similar results but noting longer execution times due to hardware limitations (Apple Mac Mini M4Pro with 32GB RAM).

Technical performance metrics demonstrate the system’s efficiency, with an average processing time of 4.5 min across 50 test runs. The workflow supports comprehensive multilingual output capabilities, enabling global application across diverse educational contexts. At SIMNOVA Simulation Center, the system has been customized to incorporate institutional branding elements, including logos and structured document templates, further enhancing the professional quality of the generated materials.

## Agentic workflow structure (n8n implementation)

The agentic workflow within n8n was designed to mimic the structure of a complex, multi-step reasoning process, drawing heavily on principles of agent-based AI systems [16].

The core concepts employed include the following:

- Decomposition (sub-agents): The overall task of scenario generation was broken down into a series of smaller, more manageable sub-tasks. Each sub-task was handled by a dedicated agent, implemented as a separate node or set of nodes within the n8n workflow. This hierarchical decomposition allowed higher-level agents to orchestrate lower-level, specialized agents, ensuring that each component of the scenario received focused attention.
- Prompt chaining (sequential execution with context passing): The workflow utilized prompt chaining extensively, where the output of one agent became part of the input prompt for the subsequent agent. This ensured that each agent had the necessary context from previous steps. This serial prompt chaining created a coherent progression of scenario elements.
- Parallelization (concurrent execution): Where appropriate, agents were executed in parallel to improve efficiency. For example, agents responsible for generating “roles and resources” and “briefing, debriefing, references” could operate concurrently, as they both depended on common inputs but were independent of each other. This parallel execution significantly reduced the overall processing time.
- Retrieval-augmented generation (RAG): The initial interaction agent utilized RAG to access and incorporate information from various knowledge sources, including a vector database populated with healthcare simulation textbooks, direct search capabilities for PubMed and Semantic Scholar, and guidelines repositories for specific clinical scenarios. This enhanced the factual accuracy and relevance of the generated content.
- Tool use (external API calls and data transformations): The workflow leveraged n8n’s ability to integrate with external APIs to perform tasks such as retrieving relevant research articles, generating text with LLMs, validating data formats, converting data between formats, and sending emails. This integration capability expanded the system’s functionality beyond what a standalone LLM could achieve.
- Iterative refinement (reviewer agent and feedback loop): A dedicated “reviewer” agent was included in the workflow to assess the output of the scenario generation process for adherence to the user’s initial query and established simulation best-practice standards. This provided a crucial layer of quality control and allowed for potential automated refinement before the final output was presented to the user.

## Detailed workflow steps and agent interactions

**Fig. 2:**
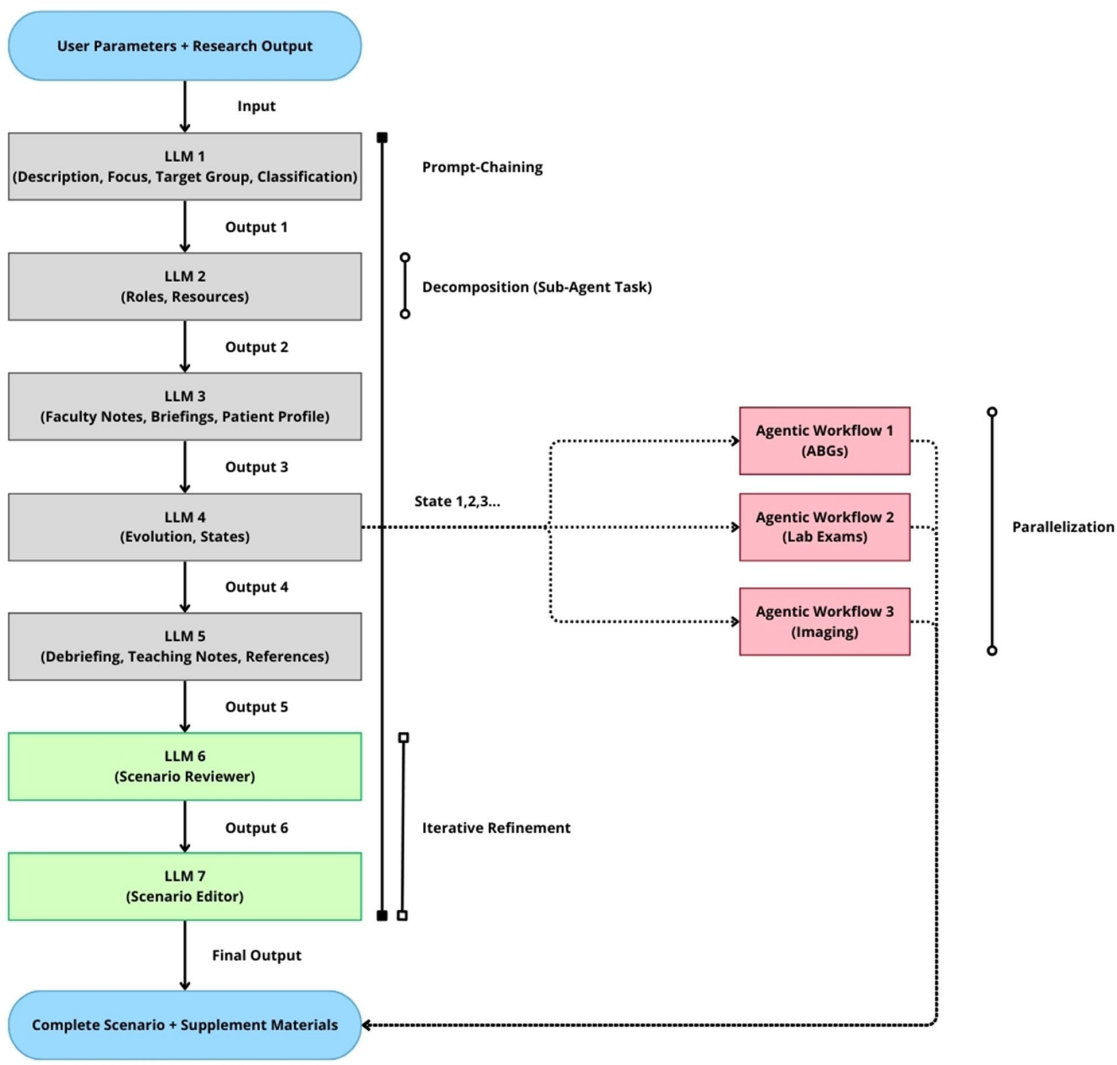
Agentic workflow for scenario generation with user feedback loop. This figure illustrates the detailed structure of the agentic AI workflow implemented in n8n for healthcare simulation scenario design. The process begins with an input comprising “user parameters + research output,” derived from the initial interaction agent’s retrieval-augmented generation (RAG) process. This input is fed into a sequence of specialized LLM agents (LLM 1 through LLM 7), each responsible for a specific sub-task of scenario generation (decomposition-sub-agent task). Prompt chaining ensures that the output of each agent informs the subsequent agent’s input, maintaining contextual coherence. The workflow demonstrates parallelization, where three separate agentic workflows generate supplementary materials (ABGs, Lab Exams, Imaging) concurrently with the main scenario components. An iterative refinement loop, involving a “scenario reviewer” agent (LLM 6) and a “scenario editor” agent (LLM 7), ensures quality control and formatting. The “final output” (complete scenario + supplement materials) is then presented back to the user for review, editing, and final approval before implementation in simulation-based education

The agentic workflow (Figs. 1b and 2) was built to operate through a conversational interface resembling familiar chat experiences, ensuring that end users were not confronted with complex coding interfaces or technical barriers. The core of the system was an initial agent with advanced retrieval-augmented generation (RAG) capabilities and access to specialized tools, serving as the front-end interface. A typical interaction began with the educator providing basic scenario requirements, providing comprehensive simulation parameters including title, main problem/diagnosis, target learner group, SimZone classification [17], simulated location, specific learning objectives, required key actions, detailed patient characteristics, preferred language output. This agent performed RAG, searching relevant databases and synthesizing findings into a comprehensive Research Output parameter that informed subsequent workflow processes. Once the educator reviewed and confirmed the parameters and the researched content, the system activated the specialized Scenario Designer n8n workflow, which orchestrated a network of interconnected AI agents:

1. Scenario outline and objectives agent: This agent received the user’s query parameters and the Research Output, generating a structured JSON object containing key elements like scenario title, description, needs assessment, learning objectives, and scenario classification metadata. The agent explicitly referenced ASPiH core values and curriculum mapping, ensuring alignment with established standards.
2. Roles and resources agent: This component generated a detailed specification of the roles required and the physical resources needed for the scenario.
3. Scenario patient and briefings creation: This agent generated structured briefings (faculty script, pre-briefing, briefing) and a detailed patient profile.
4. Scenario states agent: This component generated a structured description of the scenario states, including detailed physiological parameters, transitions between states, and resources required at each stage. The agent incorporated extensive guidance on trigger mechanisms (both time-based and action-based) and adhered to realistic clinical progressions.
5. Educational content agent: This agent focused on generating the pedagogical elements of the scenario, including desired actions for learners and facilitators, debriefing points, and relevant teaching notes and references.
6. Reviewer agent: Acting as a quality control mechanism, this agent assessed the combined output for adherence to the user’s initial query, consistency with simulation best-practice standards, clinical accuracy, completeness, and coherence. This feedback could either trigger refinements or be presented to the user as part of the final output.
7. Editor agent: This final component received all JSON data and formatted it into HTML, subsequently converted to PDF for easy distribution and printing.

In parallel with the main workflow, supplemental material creation agents generated realistic clinical documents (e.g., arterial blood gas results, imaging findings, laboratory test results) based on the patient’s condition and progression defined in the scenario states. Finally, an assembly process combined the outputs from various agents into a single, cohesive document, which was then delivered to the user’s email address.

The entire workflow represented a sophisticated orchestration of AI agents, each with specialized responsibilities yet working in concert to produce a comprehensive, standards-compliant simulation scenario. This approach not only accelerated the scenario creation process but also enhanced consistency and adherence to established guidelines.

## Scope of innovation’s potential application

The AI-driven scenario design workflow possesses a broad scope of potential application within the field of healthcare simulation. The system’s versatility allows it to be adapted to a wide range of clinical settings, including, but not limited to, emergency medicine, critical care, obstetrics, pediatrics, and mental health. Furthermore, it can be customized to suit different learner levels, ranging from undergraduate students to experienced healthcare professionals, by adjusting the complexity of the clinical presentation and the expected level of performance.

The system’s design facilitates the tailoring of scenarios to specific learning objectives, encompassing clinical skills acquisition, teamwork and communication training, and clinical decision-making practice. The inclusion of multilingual output capabilities significantly expands the system’s reach, making it applicable in diverse educational contexts across the globe. The workflow also facilitates the rapid generation of scenario variations, allowing educators to create multiple versions of a scenario with differing patient presentations, complications, or resource availability. This capability is particularly valuable for research purposes, enabling controlled comparisons of different pedagogical approaches. The generated scenarios can be used in simulation with standardized patients, and within environments such as hospital wards, simulation centers, and community settings.

## Instructions for implementation and use

Implementing and utilizing this innovation requires a series of straightforward steps. First, access to the n8n platform is necessary, either through a self-hosted instance or a cloud-based subscription. The pre-built scenario design workflow can then be imported into the n8n environment. API keys for the required AI models, such as OpenAI’s GPT models, Anthropic’s Claude, and Google’s Gemini models, must be obtained and configured within the n8n workflow.

To generate a scenario, educators provide a clear and concise brief, specifying the clinical topic, target learner level, learning objectives, and any specific requirements or constraints. The workflow is then executed within n8n, triggering the sequence of AI agent actions. Critically, after the workflow completes, a thorough review and refinement of the AI-generated scenario by a subject matter expert is essential. This step ensures the accuracy, realism, and pedagogical soundness of the scenario before it is used with learners. Finally, the finalized scenario can be implemented in simulation-based education activities.

For simulation centers with sufficient computational resources, the system can be configured to use locally-hosted, smaller LLMs rather than commercial API services. This option reduces ongoing operational costs and addresses potential data privacy concerns, making the system more accessible to resource-constrained institutions. The implementation typically requires modest technical skills comparable to those of a simulation technician or educator with basic coding experience.

## Potential pitfalls and workarounds

While the AI-driven workflow offers significant advantages, several potential pitfalls must be acknowledged and addressed. A primary concern is the potential for inaccuracies in the AI-generated content. LLMs, while powerful, do not possess true understanding and may occasionally produce information that is factually incorrect or clinically inappropriate. To mitigate this risk, a rigorous review process involving subject matter experts is crucial.

Another potential issue is bias in the AI model’s outputs, reflecting biases present in the training data. To address this, prompts were designed to encourage diverse and equitable representation, and the outputs are reviewed for inclusivity. Technical issues with the n8n platform or the AI models themselves may also arise, necessitating backup plans and access to technical support. For centers using self-hosted LLMs, additional challenges may include managing computational resources and maintaining model updates. These can be addressed through careful infrastructure planning and establishing regular update protocols. The initial learning curve for configuring n8n workflows might also present a barrier, though this can be mitigated by providing detailed documentation and establishing peer support networks among simulation centers implementing similar systems.

## Resources needed and cost estimation

The resources required for implementation include access to the n8n platform, which may involve costs depending on the chosen deployment option (self-hosted or cloud-based). API access to the chosen AI models also incurs costs, which are dependent on usage and the specific provider’s pricing structure. For centers opting for self-hosted n8n and local LLMs, initial costs would include server hardware with sufficient computational capacity, particularly if running multiple concurrent workflows.

A precise cost estimation is challenging, as it is highly dependent on usage patterns and specific pricing plans. However, it is important to consider the potential cost savings resulting from the significant reduction in scenario development time, which may offset the direct costs of the platform and API access. By our preliminary estimations, the system reduces scenario development time by approximately 70–80%, translating to substantial labor cost savings for simulation centers regularly producing new scenarios. This aligns with the findings by the University of Toronto’s pilot program, which showed reduced scenario development time by 73% while maintaining 92% clinical accuracy compared to human-authored cases when using AI as an aid to writing [18].

The self-hosted approach with local LLMs offers the most cost-effective long-term solution for centers with frequent scenario development needs, as it eliminates ongoing API fees. However, this approach requires greater initial investment in hardware and technical configuration. For centers with occasional scenario development needs, the cloud-based approach with commercial API services may offer a more flexible, pay-as-you-go solution.

The strategic use of different AI models in our implementation demonstrates how costs can be managed effectively. By utilizing Gemini 2.0 models (which are currently free to use) for the main workflow components while reserving premium models like GPT-4o and Claude 3.5 for specialized tasks requiring their unique capabilities, the system achieves cost optimization without compromising quality.

## Lessons learned

The development and implementation of this AI-assisted scenario design workflow yielded several valuable lessons. The importance of interdisciplinary collaboration between simulation experts and individuals with technical aptitude was paramount, though we demonstrated that specialized AI expertise is not a prerequisite for successful implementation. The iterative process of refining AI prompts and workflow logic proved crucial for optimizing the quality and accuracy of the outputs. The project reinforced the essential role of human oversight in AI-assisted processes, emphasizing that AI should be viewed as a powerful tool, not a replacement for human expertise. The need for user training became evident, as educators require guidance on how to effectively interact with the AI tool and interpret its outputs. Ethical considerations, particularly regarding accuracy, bias, and intellectual property, required careful attention throughout the development process. Finally, the process of creating the AI workflow unexpectedly led to a deeper understanding of our own scenario design practices, forcing us to make our implicit knowledge explicit and transferable.

Perhaps most significantly, the project demonstrated that sophisticated AI implementations in healthcare simulation are within reach of motivated clinicians and educators without requiring specialized technical expertise or external consultants. This realization has profound implications for the democratization of AI tools within healthcare education, potentially accelerating innovation and adoption across diverse settings and resource levels.

## Data Availability

No datasets were generated or analysed during the current study.

## Abbreviations

AI: Artificial intelligence
ASPiH: Association for Simulated Practice in Healthcare
INACSL: International Nursing Association for Clinical Simulation and Learning
LLM: Large Language Model
RAG: Retrieval-augmented generation
SMART: Specific, measurable, attainable, relevant, timely
